# Contribution of Mitochondrial Dysfunction Combined with NLRP3 Inflammasome Activation in Selected Neurodegenerative Diseases

**DOI:** 10.3390/ph14121221

**Published:** 2021-11-25

**Authors:** Anna Litwiniuk, Agnieszka Baranowska-Bik, Anita Domańska, Małgorzata Kalisz, Wojciech Bik

**Affiliations:** 1 Department of Neuroendocrinology, Centre of Postgraduate Medical Education, Marymoncka 99/103, 01-813 Warsaw, Poland; alitwiniuk@cmkp.edu.pl (A.L.); anita.domanska@cmkp.edu.pl (A.D.); mkalisz@cmkp.edu.pl (M.K.); wbik@cmkp.edu.pl (W.B.); 2 Department of Endocrinology, Centre of Postgraduate Medical Education, Cegłowska 80, 01-809 Warsaw, Poland; 3 Department of Physiological Sciences, Institute of Veterinary Medicine, Warsaw University of Life Sciences, Nowoursynowska 159, 02-776 Warsaw, Poland

**Keywords:** mitochondrial dysfunction, NLRP3 inflammasome, Alzheimer’s disease, Parkinson’s disease

## Abstract

Alzheimer’s disease and Parkinson’s disease are the most common forms of neurodegenerative illnesses. It has been widely accepted that neuroinflammation is the key pathogenic mechanism in neurodegeneration. Both mitochondrial dysfunction and enhanced NLRP3 (nucleotide-binding oligomerization domain (NOD)-like receptor protein 3) inflammasome complex activity have a crucial role in inducing and sustaining neuroinflammation. In addition, mitochondrial-related inflammatory factors could drive the formation of inflammasome complexes, which are responsible for the activation, maturation, and release of pro-inflammatory cytokines, including interleukin-1β (IL-1β) and interleukin-18 (IL-18). The present review includes a broadened approach to the role of mitochondrial dysfunction resulting in abnormal NLRP3 activation in selected neurodegenerative diseases. Moreover, we also discuss the potential mitochondria-focused treatments that could influence the NLRP3 complex.

## 1. Introduction

Neurodegenerative diseases (NDs) such as Alzheimer’s disease (AD), Parkinson’s disease (PD), Amyotrophic Lateral Sclerosis (ALS), Huntington’s disease (HD), and Multiple Sclerosis (MS) negatively affect millions of people worldwide and constitute an enormous burden on society. The risk of being affected by NDs increases dramatically with age. Neurodegenerative disorders are a group of congenital or acquired progressive diseases of the nervous system. The major pathological hallmarks of neurodegenerative diseases are the abnormal protein aggregation in the central nervous system (CNS). NDs occur when nerve cells of the brain or peripheral nervous system lose function and ultimately die. Although NDs are heterogeneous in their clinical images, with a variety of underlying mechanisms, mitochondrial dysfunction and systemic activation of the immune system remain common features that are implicated in the progression of these diseases [1].

Alzheimer’s disease is the most common form of dementia worldwide. It is an incurable and progressive neurodegenerative disease that leads to death. It is estimated that AD accounts for about 80% of all diagnosed cases of dementia [2]. AD was first described in 1906 by the German neuropathologist Alois Alzheimer [3]. The clinical manifestation of Alzheimer’s disease appears gradually and develops over the years. In general, the clinical features can be divided into three categories—cognitive, functional, and behavioral/psychological symptoms. From a histopathological point of view, the neuropathological hallmarks of AD include accumulation of senile plaques (SPs) and neurofibrillary tangles (NFTs) in several brain areas such as the hippocampus and frontal and parietal cortex [4]. The consequence of plaque and tangles development is loss of synapses, depletion of neurotransmitters, and, ultimately, neuronal cell death and cerebral atrophy. 

Many risk factors may contribute to AD onset. Amongst them are senescence, autophagy defects, genetic factors (i.e., ApolipoproteinaE-allele4 (APOE4), Triggering receptor expressed on myeloid cells 2 (Trem2), microbiota alterations, lifestyle, cardiovascular and traumatic brain injury, as well as environmental factors (level of education, hypertension, obesity, diabetes, smoking, hearing loss, depression, physical inactivity, social isolation) [5]. 

Parkinson’s disease (PD) is a type of neurodegenerative disease characterized by the progressive loss of dopaminergic neurons in the substantia nigra region of the brain. It is the most common neurodegenerative movement disorder. PD affects over 6 million individuals globally [6]. Two forms of family and sporadic PD are known. The family form is diagnosed in 5–10% of patients. The majority of cases, 90–95%, are patients who develop a sporadic form of Parkinson’s Disease (sPD). The most common and characteristic clinical symptoms are motor dysfunctions, including resting tremor, progressive rigidity, bradykinesia, and postural instability. Disease symptoms result directly from the progressive loss of dopaminergic neurons in the area of the substantia nigra pars compacta, which leads to a decrease in dopamine (DA) secretion in the striatum and the degeneration of dopaminergic neurons in this area [7]. A characteristic picture of degenerated neurons in PD, predominantly seen in the substantia nigra pars compacta, is the presence of Lewy bodies (LBs) containing mainly α-synuclein (SNCA) deposits in the presynaptic part of neurons. In addition, the protein aggregates include ubiquitin, α-B crystallin, APP (Amyloid Precursor Protein), MAP-5 (Microtubule Associated Protein-5), a proteasome complex, and the phosphorylated form of the IκBα protein (pIκBα) [8,9].

A large body of literature provides evidence that mitochondrial dysfunction, both primary and secondary, is increasingly recognized as a cause of neurodegeneration [10,11]. Moreover, mitochondrial disturbance can be observed even before the appearance of histopathological hallmarks of the diseases [12]. 

In recent years, mitochondria have also been described as key signaling organelles of the innate immune system. Each mitochondrion is a potent agonist of inflammation, mainly a mitochondrial DNA (mtDNA), which are normally shielded from the rest of the cell and extracellular environment and therefore do not elicit detrimental inflammatory cascades [13]. Mitochondrial damage can lead to the cytosolic and extracellular exposure of mtDNA, which triggers inflammation in neurodegenerative disorders.

There is growing evidence that mitochondrial dysfunction may activate the NLRP3 (nucleotide-binding oligomerization domain (NOD)-like receptor protein 3) inflammasome, leading to intensified local and systemic inflammation [14].

Inflammasomes are multiprotein immune complexes assembled by pattern recognition receptors (PRRs) in the cytoplasm. Its activation mediates inflammatory responses to cellular damage, pathogenic microbial infections, and various pathological states [15]. They are important components of innate immunity, and therefore, inflammasomes may be recognized as central pathogenic factors in various diseases including those of neurodegenerative origin. Indeed, the role of the best-known NLRP3 inflammasome in the pathogenesis of both Alzheimer’s and Parkinson’s disease is under intensive research.

In this review, we discuss the role of mitochondrial dysfunction in the pathogenesis of selected neurodegenerative diseases, AD and PD, in the light of over-activated NLRP3. We also present the therapeutic possibilities of targeting mitochondria and, consequently, also the inflammasome complex in AD and PD.

## 2. Mitochondria

### 2.1. Function and Structure of Mitochondria

**Figure 1:**
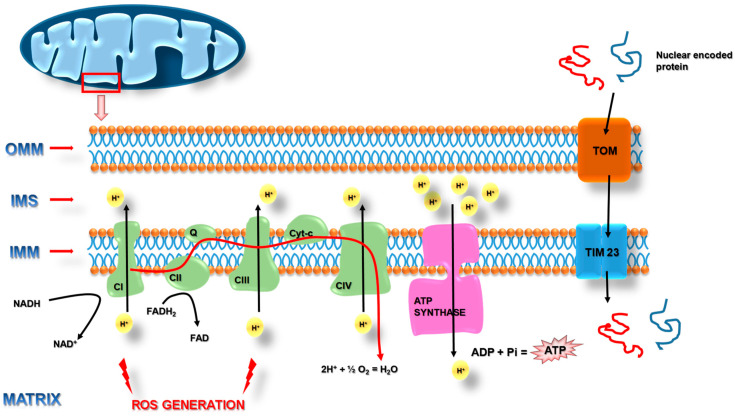
Mitochondrial electron transport chain and reactive oxygen species generation places. CI—Complex I (NADH dehydrogenase); CII—Complex II (succinate dehydrogenase); Q—Quinone; CIII—Complex III (cytochrome bc1 complex); cyt-c—cytochrome c; CIV—Complex IV (cytochrome c oxidase); OMM (outer mitochondria membrane); IMS (intermembrane space); IMM (inter mitochondrial membrane); TOM (translocase of the outer mitochondrial membrane); TIM 23 (translocase of the inner mitochondrial membrane 23). Details in the text.

Mitochondria are described as “the powerhouse of the cell”. They are also recognized as a major cellular signaling platform, with implications on cell proliferation, stem cell maintenance, cellular immunity, and cell death [16]. Mitochondria also take part in many biochemical changes in the cell, such as the regulation of calcium ion homeostasis, synthesis of hem, lipids, amino acids, or nucleotides. They are double-membrane organelles with structurally and functionally distinct outer (OMM) and inner (IMM) membranes separating the intermembrane space from the matrix [17]. The number, shape, and size of mitochondria can vary depending on cellular function [18]. Interestingly, the morphology, number, and intracellular distribution of mitochondria change during cell growth and differentiation. Mitochondrial energy production via the process of oxidative phosphorylation takes place at the IMM through the activity of respiratory chain complexes generating a mitochondrial membrane potential (mtΔΨ) that is used by the ATP-synthase enzyme complex to synthesize adenosine triphosphate (ATP) (Figure 1).

Mitochondria contain mtDNA located in the matrix. The mtDNA contains 37 genes, 13 of which encode 13 protein subunits of the respiratory chain complexes, 22 encode transfer RNAs (tRNAs), and 2 encode ribosomal RNAs (rRNAs) [19]. Other mitochondrial protein components are encoded in the nuclear DNA (nDNA) and are imported into the organelle after the translation at cytosolic ribosomes. Therefore, the maintenance of an entire and functional mitochondrial proteome requires a fine-tuned and well-coordinated sequence of many reactions as well as close integration of organellar and cellular biogenesis processes [20].

### 2.2. Mitochondrial Homeostasis

Proper mitochondrial function is maintained through the two opposing mechanisms: generation of new mitochondria by mitochondrial biogenesis, and the clearance of damaged mitochondria by mitophagy [21,22]. It should be noted that new mitochondria are generated from pre-existing forms through mitochondrial fission and do not form de novo [23].

**Figure 2:**
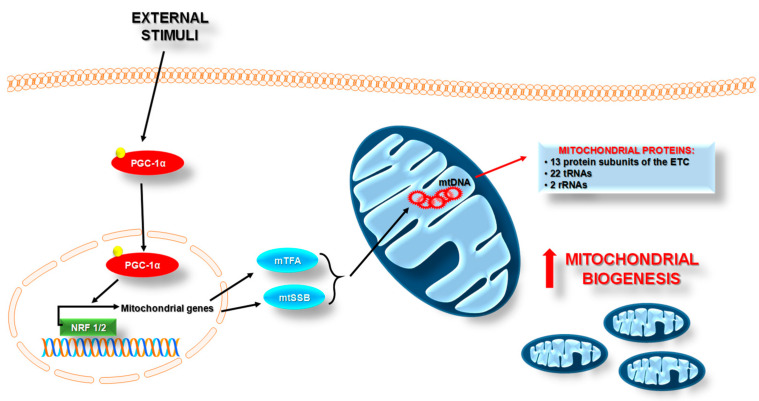
The scheme of mitochondrial biogenesis regulation. Mitochondrial biogenesis is the multistep cellular process by which cells increase the mitochondrial number. This process is activated by numerous different signals during cellular stress or in response to environmental stimuli. In response to external factors, the transcription factors are activated, especially PGC-1α (peroxisome proliferator-activated receptor-gamma coactivator-1α). PGC-1α is found mainly in the cytosol. Upon activation, it translocates to the nucleus, where it coactivates various energy metabolism-relevant transcription factors—NRF1/NRF2 (nuclear respiratory factors 1 and 2); ERR-α, -β, and -γ (the estrogen-related receptors -α, -β, and -γ); and NRF2/NFE2L2 (nuclear factor erythroid 2-related factor 2)—which then induce the expression of genes encoding proteins in electron transport chains (ETC), and along with mTFA (the mitochondrial transcription factor A) and mtSSB (the mitochondrial single-stranded DNA-binding protein), regulate the transcription, replication, and synthesis of new mtDNA.

Mitochondrial biogenesis is a complex and multistep cellular process, which includes replication, transcription, and translation of mtDNA-encoded genes, as well as loading of phospholipids and nuclear-encoded proteins in different mitochondrial subcomparments [22]. Regulation of these organelles’ biogenesis is mediated by numerous transcription factors in response to diverse stimuli, both intracellular and environmental signals (Figure 2).

The main linking factor between external stimuli and mitochondrial biogenesis is PGC-1α (peroxisome proliferator-activated receptor-gamma coactivator-1α). PGC-1α is the best-studied member of the peroxisome proliferator-activated transcriptional coactivator family that coordinates the activity of several transcription factors involved not only in mitochondrial biogenesis but also in its function [24]. In resting conditions, PGC-1α is found mainly in the cytosol. Upon activation, it translocates to the nucleus, where it coactivates various energy metabolism-relevant transcription factors. PGC-1α activates nuclear respiratory factors 1 and 2 (NRF1 and NRF2); estrogen-related receptors -α, -β, and -γ (ERR-α, -β, and -γ); and nuclear factor erythroid 2-related factor 2 (NRF2/NFE2L2). Then, these factors induce the expression of genes encoding proteins in electron transport chains (ETC), and along with the mitochondrial transcription factor A (mTFA), regulate the transcription, replication, and synthesis of new mtDNA [25].

Beyond essential roles in cell physiology, mitochondria are also the major source of reactive oxygen species (ROS) created as by-products of respiration. Most cell-derived ROS are generated at Complexes I and III (Figure 1) [26,27]. In turn, ROS oxidize proteins, lipids, and nucleic acids, inside and outside of the mitochondria, leading to mitochondrial malfunction and cellular damage. Noticeably, mtDNA is not protected by histones and its mutation rate is 10 times higher than that of nuclear DNA [28]. Furthermore, it was observed that during aging, mtDNA can be damaged by exposure to ROS, which is generated during the oxidative phosphorylation process. Mutations in mtDNA, both through inheritance or somatic accumulation, can compromise mitochondrial function and result in cell death and the progression of neurodegenerative diseases. In addition, it was described that mitochondria are recognized as a source of damage-associated molecular patterns (DAMP). Particularly, mtDNA, mTFA, ROS, cytochrome c, and cardiolipin (CL), after being released from mitochondria into the cytosol, can induce inflammatory responses [29,30].

**Figure 3:**
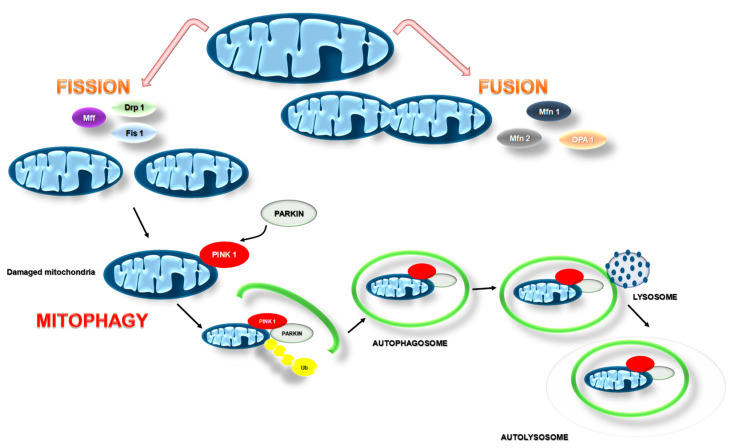
The scheme of mitochondrial dynamics and mitophagy. Mitochondrial fusion and fission processes are responsible for a healthy mitochondrial network through the segregation and subsequent degradation of damaged mitochondria. Mitochondrial fusion is a two-step process. The fusion of the outer mitochondrial membrane is regulated by Mfn1 (Mitofusin 1) and Mfn2 (Mitofusin 2), whereas fusion of the inter mitochondrial membrane is mediated by OPA1 (optic dominant atrophy 1) and mitochondria-specific cardiolipin (CL). Mitochondrial fission is mediated predominantly by Drp1 (a dynamin superfamily GTPase) and recruited to scission sites by resident outer-membrane proteins including Mff (mitochondrial fission factors) and Fis1 (mitochondrial fission 1 protein). The fission process generates fragmented mitochondria, which are conducive to the sequestration of injured fragments for subsequent degradation through mitophagy. PTEN-induced kinase 1 (PINK1) and the Parkin pathway regulate the initiation of mitophagy. Mitochondrial damage results in the accumulation of PINK1 on the outer mitochondrial membrane, where it phosphorylates polyubiquitin chains linked to mitochondrial outer membrane proteins. Next, phospho-S65-ubiquitin binds to Parkin, recruiting it from the cytosol and activating Parkin’s E3 ubiquitin ligase activity. This complex is identified by ubiquitin-SQSTM1(multifunctional adaptor protein sequestosome 1)-LC3 (microtubule-associated protein 1 light chain 3) autophagosome targeting and is degraded following lysosomal fusion.

The next critical pathway of mitochondrial quality control is mitophagy, which allows eukaryotic cells to remove damaged mitochondria and preserve mitochondrial health. The process of mitophagy occurs under harmful conditions, such as oxidative stress, hypoxia, mitochondrial transmembrane potential loss, accumulation of unfolded proteins, and iron starvation [31]. In eukaryotic cells, several pathways of mitophagy regulation have been described, but the best-studied pathway is mediated by the phosphatase and tensin homolog (PTEN)-induced putative kinase 1 (PINK1) and the E3-ubiquitin ligase Parkin (Figure 3).

In non-stressful conditions with normal mitochondrial membrane potentials, PINK1 is imported via the translocase of the outer membrane and translocase of the inner membrane (TOM/TIM) complex in a membrane potential-dependent manner into mitochondria, where the PINK1 is cleaved by mitochondrial processing peptidase (MPP) and presenilin-associated rhomboid-like protease (PARL) [32,33]. Then, the cleaved PINK1 is released to the cytosol and degraded by the proteasome [34]. In unhealthy mitochondria, the inner mitochondrial membrane becomes depolarized. Upon depolarization of the mitochondrial membrane potential, the cleavage of PINK1 is abolished, and PINK1 is stabilized on the outer mitochondrial membrane. In unhealthy mitochondria, PINK1 with kinase activity regulates the recruitment and activation of the cytosolic Parkin via direct phosphorylation of the Parkin Ub-like (UBL) domain or the phosphorylation of ubiquitin. Once active, Parkin ubiquitinates numerous substrates at the outer mitochondrial membrane, including mitofusin1 (MFN1), mitofusin2 (MFN2), translocase of outer membrane20 (TOM20), mitochondrial Rho GTPase1 (Miro1), and voltage-dependent anion-selective channel 1 (VDAC1) [35]. In 2019, Bernardini and co-workers reported that the apoptotic protein BAK is a Parkin target, further connecting Parkin-mediated mitophagy to the regulation of cellular apoptosis [36]. Through controlling these mitochondrial proteins, the PINK1–Parkin pathway plays a crucial role in maintaining mitochondrial homeostasis by regulating mitophagy, mitochondrial dynamics, and mitochondria-mediated apoptosis.

Mitochondrial fusion and fission are also extremely important processes in the regulation of mitochondrial homeostasis. Besides mitophagy, mitochondrial dynamics ensure a healthy network through the segregation and subsequent degradation of damaged mitochondria. Mitochondrial dynamics indicate whether damaged mitochondria are to be repaired or degraded by mitophagy [37]. The fission and fusion processes are mainly regulated by proteins that belong to a dynamin-related family of large GTPase, including conserved dynamin-related GTPase (Drp1), conserved dynamin-related GTPase mitofusin 1 and 2, and optic dominant atrophy 1 (OPA1) [38]. Mitochondrial fusion is a two-step process. The fusion of OMM is regulated by MFN1 and MFN2, whereas fusion of the IMM is mediated by OPA1 and mitochondria-specific cardiolipin [39]. In turn, outer membrane fission is mediated predominantly by a dynamin superfamily GTPase, Drp1, that is localized to the cytoplasm and recruited to scission sites by resident outer-membrane proteins including Mff (Mitochondrial fission factor), Fis1 (Mitochondrial fission 1 protein), and mitochondrial dynamic proteins 49 and 51 (Mid49 and Mid51) [40].

As it has been shown above, mitochondria could be considered as an integrated, subcellular system with a complex, highly dynamic transport pathway that is essential for proper cell homeostasis. Thus, disruption of the above systems can lead to mitochondrial dysfunction and may serve as a central mechanism of many diseases, including neurodegenerative diseases.

## 3. Inflammasomes

### 3.1. Inflammasomes and Mitochondria

Inflammasomes are a group of intracellular complexes located in the cytosol, which are an element of non-specific immunity, responsible for the detection of molecular patterns indicative of both DAMPs and the presence of pathogen-associated molecular patterns (PAMP) and production of pro-inflammatory cytokines [41]. To date, the best-characterized inflammasomes are NLRP1 (nucleotide-binding oligomerization domain (NOD)-like receptor protein 1), NLRP2 (NOD-like receptor protein 2), NLRP3, NLRC4 (CARD domain-containing protein 4, also called IPAF (ICE-protease activating factor)), and AIM 2 (absent in melanoma 2) inflammasome [42]. These high-molecular-weight complexes play a role in the activation, maturation, and release of the pro-inflammatory cytokines, interleukin 1β (IL-1β), and interleukin 18 (IL-18).

The inflammasome complex is composed of a pattern recognition receptor acting as the sensor molecule, an adaptor apoptosis speck protein (ASC), and pro-caspase-1 as the effector molecule. NLRP3 is the best-studied member of the inflammasome family. It has been revealed that under normal physiological conditions, the activity of this inflammasome is extremely low to maintain a low inflammatory state [15]. However, activation of NLRP3 results in the discharge of inflammatory factors, including IL-1β and IL-18. The process of activation requires two distinct, independent steps, which are priming and activation. The first signal is started when PAMPs and/or other inflammatory mediators (IL-1β and tumor necrosis factor α (TNF-α)) bind to their respective receptors (Pattern Recognition Receptors IL-1βR and TNF-αR) [43] that induce the activation and translocation into the nucleus of nuclear factor kappa-light-chain-enhancer of activated β cells (NF-κβ). In turn, the transcription of NF-kβ-dependent genes such as NLRP3, pro-IL-1β, and pro-interleukin-18 (pro-IL-18) is promoted [44,45,46]. The activation signal includes extracellular stimuli such as tissue damage, metabolic dysregulation, ATP, cholesterol, uric acid, and amyloid β, which result in the release of ROS, as well as lysosomal enzyme cathepsins B or L and Ca^2+^ caused by destabilization and rupture of lysosome or endosome [47,48]. As a consequence of disturbed ionic flux, mitochondrial dysfunction may occur. The characteristic features of mitochondrial dysfunction comprise the increase in mitochondrial ROS production, oxidized mitochondrial DNA release to the cytosol, dysregulation of mitochondrial dynamics, and impaired mitochondrial membrane potential [49,50].

As described in the literature, mitochondria-generated ROS and oxidized mtDNA serve as DAMPs. They could trigger NLRP3 inflammasome formation and activation [51,52]. In addition, cardiolipin, a mitochondria-specific phospholipid located physiologically in the inner membrane of mitochondria, after translocation to the outer membrane promotes the oligomerization of NLRP3 [53].

The process of NLRP3 activation initiates a cascade of events that results in the discharge of inflammatory factors (including IL-1β and IL-18), cell swelling, membrane rupture, and eventually triggering a lytic, pro-inflammatory form of cell death, termed pyroptosis [52,54,55,56].

Therefore, it could be stated that mitochondria play an important role in the NLRP3 inflammasome activation.

### 3.2. NLRP3 Inflammasome and AD

NLRP3 inflammasome activation has been implicated in the pathogenesis of AD [1,57]. It must not be forgotten that mitochondrial dysfunction also plays an important role in the pathological processes occurring in AD.

**Figure 4:**
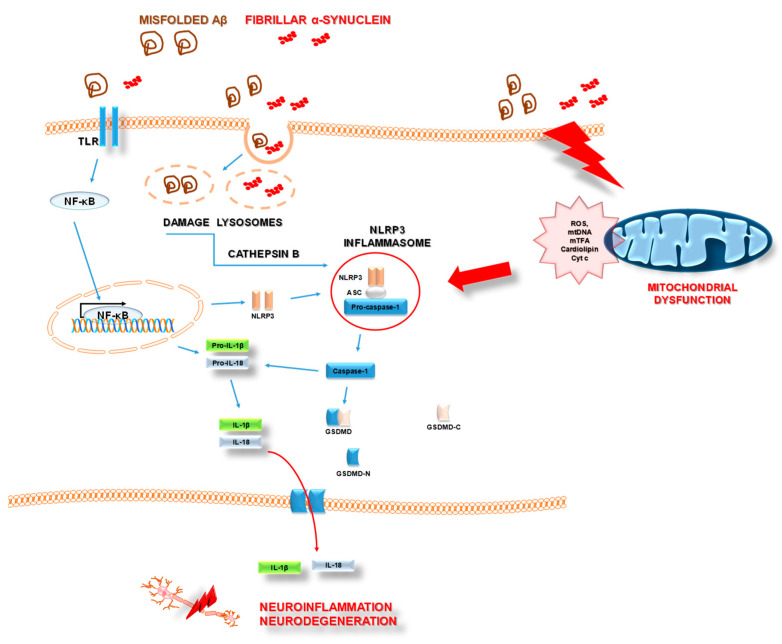
The scheme of the signaling cascade associated with NLRP3 inflammasome activation in Alzheimer’s disease and Parkinson’s disease. Aβ (Amyloid β); TLR (Toll-Like Receptor); NF-κβ (Nuclear Factor kappa-light-chain-enhancer of activated β cells); IL-1β (Interleukin 1β); IL-18 (Interleukin 18); NLRP3 (nucleotide-binding oligomerization domain (NOD)-like receptor protein 3); ASC (adaptor apoptosis-associated speck-like protein containing a CARD); GSDMD (Gasdermin D); ROS (reactive oxygen species); mtDNA (mitochondrial DNA); mTFA (Mitochondrial transcription factor A); Cyt c (Cytochrome c). Details in the text.

Misfolded β-amyloid (Aβ), which is the key histopathological feature of AD, acts as a second signal activator of the NLRP3 complex (Figure 4). Aβ-mediated NLRP3 activation causes IL-1β secretion in a mechanism of NLRP3, ASC, and caspase-1 activities and requires cathepsin B released from damaged lysosomes [1,58]. All these events drive microglia recruitment and result in neuroinflammation. In detail, abnormal microglia-specific NLRP3 activation induces microglia Aβ phagocytic dysfunction, peripheral nerve cell damage, and other severe pathological processes, including the promotion of Aβ plaque formation and accumulation [59,60]. Furthermore, inflammatory response due to excessive NLRP3 activation and elevated IL-1β levels in microglia may result in concomitant and intensified neural tau hyper-phosphorylation, neurofibrillary tangles, and synaptic dysfunction in AD [60].

The interaction between the NLRP3 inflammasome and AD pathology is confirmed by the findings from clinical and experimental studies. Increased gene expression of NLRP3, ASC, caspase-1, and pro-inflammatory cytokines IL-1β and IL-18 [61]. Furthermore, exacerbated caspase-1 activity was shown in brain lysates obtained from AD individuals. An animal transgenic model of AD, amyloid precursor protein (APP)/presenilin (PS1) mutant mice, has enhanced active caspase-1 levels [1].

It could be stated that in AD, the NLRP3 inflammasome is a part of a vicious cycle that includes pathological factors and neuroinflammation (Figure 4). 

### 3.3. NLRP3 Inflammasome and PD

Recent studies have also indicated a role of the NLRP3 inflammasome in PD. According to the current knowledge, three features of PD can be distinguished: neuroinflammation, the gradual loss of dopaminergic neurons in the substantia nigra and striatum, and concomitant accumulation of α-synuclein.

The process of neuroinflammation may be related to activated microglial NLRP3 inflammasomes [62]. Activation of NLRP3 could be triggered by the presence of fibrillar α-synuclein and the absence of α-synuclein-mediated dopaminergic neurons [63]. Findings of increased levels of cleaved caspase-1 and ASC in the substantia nigra of PD individuals as well as expression of NLRP3 in activated microglia in post-mortem tissue lysates confirm the link between neuroinflammation in a course of PD and activated NLRP3 inflammasome [62,63].

Abnormal aggregates of α-synuclein were reported to activate the NLRP3 inflammasome of microglia through interaction with toll-like receptors (TLRs). Of note, misfolded α-synuclein acts as a DAMP and alters microglial TLR expression. Then, pro-inflammatory cytokines are released through the translocation of NF-κβ. Finally, impairment of mitochondria occurs, leading to dopaminergic neuron damage [62].

To summarize, the activation of NLRP3 inflammasomes, α-synuclein aggregation, and mitochondrial dysfunction are all important factors in sustaining persistent neuroinflammation and neuronal cell death (Figure 4).

## 4. Mitochondrial Dysfunction in Neurodegenerative Disease

Neurons have high energy requirements; therefore, properly functioning mitochondria are necessary for membrane remodeling, synaptic spine formation, and the generation of transmembrane resting and action potentials in neurons [64]. In nerve cells, a large proportion of the ATP pool is used to maintain and renew the ion gradients that change during intracellular signaling, and for the uptake and transformation of neurotransmitters [65]. Thus, the presence of healthy mitochondria in nerve endings is essential for neurons to communicate with neighboring cells. In addition, the strictly defined location of the mitochondria in the neuron is also crucial to regulate changes in local concentrations of calcium ions. Previous studies showed that mitochondrial calcium ions can promote ATP synthesis by activating multiple components of the tricarboxylic acid cycle (TCA) and OXPHOS supercomplexes in many cell types, including in neurons [66,67].

Therefore, it is not surprising that mitochondrial dysfunction can have negative effects on the differentiation and survival of neuron cells. Neurons are usually unable to regenerate, so in case of damage or death, they cannot be replaced. This phenomenon can lead to the development of neurodegenerative diseases. In the pathogenesis of neurodegenerative diseases, both acute (e.g., ischemic stroke, mechanical injuries of the brain and spinal cord) and chronic (e.g., Alzheimer’s disease, Parkinson’s disease, Huntington’s disease, Amyotrophic Lateral Sclerosis), the role of mitochondrial dysfunctions is emphasized as it leads in the final stage to the generation of apoptotic signals and the death of nerve cells [68,69,70,71]. In addition, the process of aging and occurrence of misfolded proteins increases the risk of intensification of mitochondrial ROS production, accumulation of the mtDNA mutations, alteration of mitochondrial mass, compromised mitochondrial functions, chronic immune activation, and accelerated cell death [72].

### 4.1. Mitochondrial Dysfunction in Alzheimer’s Disease

It should be highlighted that several recent studies have demonstrated a connection between mitochondrial dysfunction and AD. Moreover, mitochondrial dysfunction was observed in AD postmortem brains, in platelets derived from AD patients, in AD transgenic mice, and in cell lines that express mutant APP and/or cells treated with Aβ [49,73]. A wide spectrum of relationships between mitochondrial dysfunction and the hallmarks of AD are summarized below.

#### 4.1.1. Deficiency in Mitochondrial Oxidative Phosphorylation in AD

The brain is one of most the high-energy consuming organs, consuming 20% of total body energy expenditure in a resting awake state. Therefore, neuron cells are exquisitely sensitive to any disruption or reduction in ATP production by the OXPHOS system. Impaired mitochondrial OXPHOS efficiency, dysfunction of mitochondrial respiratory enzymes, and decreased mitochondrial complex (I–V) activities were detected in the AD brain and various AD models [69]. Moreover, the clinical study of bioenergetics profiles of fibroblasts isolated from patients with late-onset AD (LOAD) and healthy controls indicated that cells from LOAD patients present the metabolic shift from the mitochondrial oxidative phosphorylation system to glycolysis, which indicate reduced mitochondrial metabolic potential (OXPHOS) in LOAD [74]. A direct effect of Aβ on the respiratory chain has also been shown. In consequence, disturbances of ETC function occur, although the exact mechanism remains unclear. A large body of evidence indicates that in AD brains, the activities of the enzymes involved in mitochondrial energy production, such as Complex IV cytochrome c oxidase (COX), pyruvate dehydrogenase complex, α-ketoglutarate dehydrogenase (αKGDH), mitochondrial isocitrate dehydrogenase, and ATP synthase complex were decreased, whereas activities of the succinate dehydrogenase (Complex II) and malate dehydrogenase were increased [75,76,77]. The dysfunction of the mitochondrial enzymes consequently compromises the maintenance of the mtΔΨ and decreased mitochondrial ATP production [78]. Beck and co-workers reported a reduction in synaptic mitochondrial Fo-ATP synthase activity by 67% in an AD mouse model compared to control groups. They also observed that decreased ATP-synthase activity resulted in a reduction in ATP, increased oxidative stress, and reduced ΔΨm due to the opening of the mitochondrial permeability transition pore (mPTP), which leads to the release of apoptotic effectors into the cytoplasm [78]. In agreement with the previous findings, our research also described that Aβ treatment decreased mitochondrial activity and mtΔΨ in differentiated SH-SY5Y cells, which consequently led to the death of neuronal cells [49]. In an animal study, Carvalho and co-workers observed that mitochondria isolated from triple transgenic AD model mice (3xTg-AD) brains have a decrease in mtΔΨ, ATP/ADP ratio and an impairment of the respiratory activities [79]. In another study, the Zhang team revealed that the brain tissue of APP/PS1 AD model mice contained fewer ATP contents compared to the wild-type mice brain samples from 5-month-old animals [80].

However, the question could be raised of how Aβ accumulates inside the mitochondria. It is known that both the amyloid-β protein precursor (AβPP) and Aβ accumulate within the mitochondria of AD brain tissue [81,82,83]. It was shown that both AβPP and Aβ were deposited in the mitochondrial protein import channel of the human AD brain, forming protein complexes with the translocase of the outer mitochondrial membrane (TOM) and with the translocase of the inner mitochondrial membrane 23 (TIM23) [83,84]. This accumulation of AβPP in the mitochondrial membrane translocases could prevent the normal import of nuclear-encoded OXPHOS proteins, including components of Complex IV, which in turn reduce COX activity [85].

Damage to the OXPHOS machinery not only hinders sufficient ATP production, but also increases the production of free radicals, which in turn damage mitochondrial proteins, influence mPTP activation, and lead to mtDNA mutagenesis, further contributing to the deterioration of the OXPHOS system.

#### 4.1.2. Disruption of Mitochondrial Homeostasis in AD

With age, the capacity of mitochondrial biogenesis declines and consequently increases the risk of the development of neurodegenerative disease. Many researchers have reported changes in the expression of transcription factors that are associated with the regulation of mitochondrial biogenesis in the brain of AD patients and animal models of amyloidosis. Gong and colleagues in their study found that the expression of PGC-1α was significantly decreased in the brains of aging Tg2576 mice (the mice overexpressing APP with the familiar Swedish mutation) [86]. Reduced expression of PGC-1α was also observed in APP/PS-1 double transgenic (2xTg-AD) mice [87]. Moreover, Wang and co-workers found that overexpression of PGC-1α, caused by an adeno-associated virus (AAV), in the hippocampus of 2xTg-AD mice resulted in a significant reduction in the expression of Aβ plaques and 8-oxo-dG [87]. The Qin team made similar observations on human post-mortem brain (hippocampal formation) samples from AD cases [88]. For the first time, they described that PGC-1α expression was decreased in the brain of AD individuals, reflecting dementia severity. In turn, Oka and colleagues focused on the mitochondrial biogenesis of PGC-1α effectors downstream, such as mTFA. They examined the effects of human mitochondrial transcriptional factor A (hmTFA) on the pathology of a mouse model of AD (3xTg-AD) [89]. In the study, the authors observed that increased expression of hmtTFA in 3xTg-AD significantly improved cognitive function, reduced oxidative stress and intracellular Aβ, and increased expression of transthyretin known to inhibit Aβ aggregation. Recently, in the AD mouse model (mutant human transgenes of APP and Presenilin-1 (PS1)), the mitochondrial biogenesis markers (PGC-1α, NRF1 and 2, and mTFA) were also declined, particularly in the hippocampus region [90]. Based on the above results, it could be suggested that therapeutic preservation of neuronal PGC-1α expression and its downstream effectors can prevent the generation of amyloidogenic Aβ peptides. 

In the early stage of AD, studies have also revealed that there is a greater increase in Drp1 compared to Mfn2 expression, which results in disturbed mitochondrial dynamics. As mentioned above, mitochondria are highly dynamic organelles that are continually altering morphology by rapid and reversible fission and fusion. The fission and fusion processes are essential for cell survival and are strongly linked to mitochondrial signaling via reactive oxygen or nitrogen (RNS) species. Neurons affected by AD present unbalanced mitochondrial dynamics, which results in structural and functional abnormalities leading to neuronal damage [91,92]. In studies using electron microscopy, dysfunctional mitochondrial morphology was observed in AD brains [93]. Unbalanced mitochondrial dynamics (increased mitochondrial fission and decreased fusion) may explain the increased mitochondrial fragmentation and defective mitochondrial function observed in neurons affected by AD. In in vitro models of AD, it was illustrated that overexpression of APP or Aβ treatment causes profound fragmentation of mitochondria with elevated Drp1 levels and altered distribution of mitochondria, which likely trigger Aβ-induced synaptic defects in neuronal cultures [49,94]. Increased mitochondrial fission is also well documented in AD patients and animal models of AD [95,96]. The feasibility of mitochondrial dynamics could be another treatment target for neurodegeneration in AD. 

Under normal conditions, when mitochondria become damaged, mitophagy is activated. It is a selective type of autophagy, by which faulty mitochondria are sequestrated into autophagosomes for subsequent lysosomal degradation. It has been shown that in the initial stage of the disease, mitophagy is activated to protect neuronal cells from death. In our previous study, we showed that Aβ treatment significantly increased mRNA levels of PINK-1 and PARKIN in differentiated SH-SY5Y cells [49]. Similar results were presented by Ye and colleagues, as the authors showed that the Parkin pathway is robustly induced upon progressive Aβ accumulation and mitochondrial damage in both human patient brains and animal models of AD [97]. However, over the disease progression, the cytosolic Parkin is depleted in AD brains, resulting in mitophagy pathology and augmented mitochondrial defects. Electron microscopy studies showed the accumulation of damaged mitochondria, characterized by the swollen appearance and distorted cristae in biopsy of human AD cases and transgenic animal models of AD [98,99]. Another study by Fang and co-workers indicated that the levels of mitophagy-associated proteins Bcl_2_L_13_, PINK1, and BCL2/adenovirus E1B 19 kDa protein-interacting protein 3-like (BNIP_3_L/NIX) were reduced and mitophagy initiation proteins such as phospho-ULK1 (Ser555) and phospho-TBK1 (Ser172) were inactivated in AD patient samples [100]. Martin-Maestro with a team showed a reduction in PINK1 and Parkin translocation to damaged mitochondria in APP- and tau-overexpression models, suggesting the role of compromised mitophagy in the accumulation of damaged mitochondria in AD models [101]. Taken together, the above results indicate that defective mitophagy is likely involved in AD-associated neurodegeneration, and that pharmacological reinstallation of mitophagy could mitigate amyloid and tau pathologies.

#### 4.1.3. Mitochondrial Dysfunction and Neuroinflammation in AD

Mitochondrial dysfunction and neuroinflammation are observed in AD. It has been noted that mitochondrial dysfunction and inflammation are interdependent lesions. Mitochondrial dysfunction and its associated oxidative stress and inflammation are increasingly appreciated as common features of neurodegeneration. The dysfunction of both organellar and molecular levels of mitochondria homeostasis mechanisms leads to irreversible damage of mitochondria and consequent release of its components, ROS, mtDNA, mTFA, membrane lipid cardiolipin, and proapoptotic proteins (including cytochrome c), into the cytosol. In turn, the leakage of mitochondrial components can act as intrinsic danger-associated molecular patterns (DAMP). Then, it could activate the oligomerization domain-like receptor family members 3 (NLRP3 inflammasome complex), which in turn boosts the production of cytokines such as IL-18 and IL-1β and induces pyroptosis in cells [14]. 

As mentioned before, NLRP3 inflammasome activation has been implicated in the pathogenesis of AD. 

It has been described that mtDNA induces neuroinflammation in vivo. Wilkins et al. showed that injection of mitochondrial lysates or mtDNA into the hippocampal dentate gyri triggered the pro-inflammatory signaling in the mouse brain, suggesting that mitochondria-derived DAMP molecules can also influence AD-associated biomarkers [102]. These authors also observed that hippocampal injection of extracellular whole mitochondria lysates or mtDNA led to NF-κβ phosphorylation, induction of TNFα mRNA, and a decrease in TREM2 (Triggering receptor expressed on myeloid cells 2) expression, all of which are closely associated with AD pathology. The authors showed that mitochondrial lysates also upregulated endogenous APP and Aβ. These findings strongly support the correlation between mtDNA and AD pathology.

Another pro-inflammatory factor released from damaged mitochondria is mTFA. It was observed that this factor could also play a role in the CNS inflammation observed in neurodegenerative diseases. In an in vitro study, Little and colleagues showed that mTFA applied at low μg/mL concentrations to human microglia-like THP-1 monocytic cells and peripheral blood monocytes increased the expression of IL-1β, Interleukin 6 (IL-6), and Interleukin 8 (IL-8) [103]. They also observed the decreased viability of human SH-SY5Y neuronal cells exposed to supernatants from monocytic cells treated with mTFA and IFN-γ (Interferon γ). In another study, Schindler et al. indicated that injection of mTFA into the cisterna magna of male Sprague–Dawley rats triggered neuroinflammation in the hippocampus and frontal cortex, which are the predominant brain areas affected by neurodegeneration in AD [104]. Better understanding the mechanisms by which molecules released from damaged mitochondria act may result in an indication of therapeutic targets in the treatment of AD.

### 4.2. Mitochondrial Dysfunction in Parkinson Disease

The pathophysiology of PD is complex and remains poorly understood, but growing evidence implicates mitochondrial dysfunction as a possible primary cause for cell death in PD [105,106]. Mitochondrial dysfunction in PD can result from several causes, including impairment of mitochondrial biogenesis, increased ROS production, mtDNA mutation, defective mitophagy, compromised trafficking, OXPHOS dysfunction, imbalanced mitochondrial dynamics, and calcium (Ca2+) imbalance, which we discuss below.

#### 4.2.1. Deficiency in Mitochondrial Oxidative Phosphorylation in PD

Parkinson’s disease is associated with a decrease in the activity level of the mitochondrial Complex I (NADH dehydrogenase). Complex I is a key entry point for electrons into the ETC and is responsible for approximately 40% of mitochondrial ATP production [107]. This complex can be damaged both as a result of the action of free radicals and the wrong folding of proteins that make up its subunits. Moreover, inhibitors of Complex I such as MPP+ (1-methyl-4-phenylpyridinium), rotenone, 6-hydroxydopamine, and annonacin all induce PD-like phenotypes, suggesting that mitochondrial dysfunction is sufficient to promote neuronal dysfunction in PD [108,109,110]. The action of these toxins lowers ATP levels and induces the production of ROS, resulting in oxidative stress. Damage to Complex I of the respiratory chain is observed in PD patients, both in the mitochondria of the dopaminergic neurons of the substantia nigra, as well as in lymphocytes, platelets, and skeletal muscle tissue [111]. The dysfunction of Complex I reducing the activity Complexes II and III in ETC and mTFA have also been reported in PD patients [112]. These changes lead to ROS production and cell death.

#### 4.2.2. Disruption of Mitochondrial Homeostasis in PD

In 2006, Barsoum and colleagues presented for the first time the results of research conducted on live dopaminergic neurons in which changes in the dynamics of mitochondria under the influence of oxidative stress, not related to mutations in nuclear DNA, were determined [113]. Their studies on the rat dopaminergic N27 neuron line showed that the inhibition of Complex I by the administration of MPP+ and the related oxidative stress led to fragmentation of the mitochondrial network in the whole cell. In recent studies, Wang et al. indicated that SNCA has been shown to influence mitochondrial size both independently and dependent on fusion/fission proteins [114]. It was also described that oligomeric SNCA can bind to lipids in the outer mitochondrial membrane and disturb the mitochondrial membrane curvature, and these processes result in a decrease in the mitochondrial fusion rate [115]. Moreover, the overexpression of SNCA in transgenic mice decreased Mfn1 and Mfn2 protein levels, correlating with a decrease in mitochondrial fusion and smaller mitochondria [116]. Other authors observed decreased levels of OPA1 in the substantia nigra of patients with sporadic PD, suggesting mitochondrial fusion deficiency [117].

Mitochondrial dynamics also include clearance of damage mitochondrial by mitophagy. Interestingly, mutations in PINK1 and Parkin are the most commonly known causes of autosomal recessive and early-onset PD, which may occur before the age of 45 [118]. As a result of the mutation of the pink1 gene, both damaged and old mitochondria that are the source of a large number of free radicals cannot be degraded. In consequence, these phenomena cause neuronal dysfunction or death, leading to PD symptoms. In studies using the PINK1-mutant Drosophila model, researchers observed a change in the morphology of the mitochondria, reduced level of ATP, shortened life span, and decreased motor activity [119]. Parkin gene mutation results in a phenotype similar to that observed in mutant PINK1. Drosophila melanogaster Parkin gene mutants show a decrease in the amount of ATP, degeneration of dopaminergic neurons, and a defect in the morphology of the mitochondria [120]. The important role of Parkin mutations in mitophagy impairment was also confirmed in an in vitro study using a specific induced pluripotent stem cell (iPSC)-derived dopaminergic neurons with mutations in the Parkin gene [121]. Another study confirming the role of mitophagy in PD found that SYNC overexpression decreases the level of microtubule-associated proteins 1A/1B light chain 3B (LC3)-positive vesicles in human neuroblastoma cells [122].

#### 4.2.3. Mitochondrial Dysfunction and Neuroinflammation in PD

Similar to AD, innate immunity-mediated neuroinflammation actively contributes to the onset and progression of PD. Recent studies have also indicated that classic neurotoxin-induced models of PD (described above) activate NLRP3 inflammasomes in the brain and peripheral organs such as the thymus [123,124,125]. Two signals are involved in the NLRP3 activation: priming and activation. Lee and colleagues described that MPTP acts as a priming signal for the NLRP3 inflammasome activation [125]. Moreover, it is known that neurotoxins function by damaging mitochondria, and they might indirectly activate NLRP3 inflammasomes by increasing ROS production. As we mentioned above, damaged mitochondria play a crucial role in the activation of the NLRP3 inflammasome by releasing mitochondrial DAMPs, such as mitochondrial ROS and mtDNA. In a clinical study, von Herman et al. indicated that in human post-mortem brain from PD cases NLRP3 expression was elevated in mesencephalic neurons [126]. They also observed that NLRP3 genetic polymorphisms were associated with the downregulation of NLRP3 activity and reduced risk of PD. Another report demonstrated that the fibrillar form of α-synuclein induced caspase-1-mediated IL-1β secretion in human monocytes and BV2 microglial cells [127]. 

Additionally, Gordon et al. indicated that the inhibition of inflammasomes by using a small-molecule NLRP3 inhibitor MCC950 prevented SYNC pathology and neuron degeneration [63]. 

Interestingly, a study about the role of mitophagy in innate immunity revealed that PINK and Parkin could act as inflammatory suppressors and that PINK-Parkin-mediated mitophagy can restrain innate immunity and alleviate the inflammatory phenotype [128]. The disturbance of mitophagy observed in PD leads to the production of damaged mitochondria and the release of ROS, mtDNA, cytochrome c, etc., to the cytosol. Then, these molecules, as DAMPs, activate the inflammasome complex. Neurotoxins, aggregated α-synuclein, mitochondrial ROS, and dysregulated mitophagy are all key regulators of NLRP3 inflammasome activation that result in IL-1β and IL-18 release, as well as pyroptotic cell death of neurons in the substantia nigra (SN) [129,130].

## 5. Mitochondria as a Therapeutic Goal in Neurodegenerative Disease Treatment

As we mentioned above, mitochondrial dysfunction plays a critical role in neurodegenerative disease. Mitochondrial dysfunctions involved in the pathophysiology of AD and PD include disturbances in OXPHOS, which in turn result in increased release of ROS, mtDNA, cytochrome c, imbalanced mitochondrial dynamic, failure mitophagy, and, consequently, the induction of inflammation. Therefore, growing attention has been paid to the search for compounds that will decrease oxidative stress and restore the proper functioning of the mitochondria. 

### 5.1. Enhance Electron Transport Chain Activity

Compounds that exert their function through interaction with the respiratory chain component belong to one of the widest classes of mitochondria-targeted therapeutic agents. Among these, some of the most promising ones are the antioxidant molecules 10-(6′-plastoquinonyl decyltriphenyl-phosphonium (SkQ1), the mitochondrial-targeted antioxidant mitoquinone MitoQ, MitoTEMPOL, and MitoVitE, which prevent apoptosis by mitigating the oxidative damage [131]. The MitoQ is known to cross the inner mitochondrial membrane and accumulate within mitochondria and, unlike other antioxidants, to reduce ROS levels generated in mitochondria. Jeong et al. examined the effects of treadmill exercise (TE) and MitoQ, individually or combined, on learning and memory, mitochondrial dynamics, NADPH oxidase activity, and neuroinflammation and antioxidant activity in the hippocampus of D-galactose-induced aging rats [132]. The authors observed that TE alone and TE combined with MitoQ in aging rats reduced mitochondrial fission factors (Drp1, Fis1) and increased mitochondrial fusion factors (Mfn1, Mfn2, OPA1). In the TE combined with MitoQ rats, they also observed improvement of NADPH oxidase and antioxidant activity (SOD-2, catalase). 

Additionally, coenzyme Q10 (CoQ10), curcumin, vitamin E, Gingko Biloba, lipoic acid, and melatonin possess mitochondrial restoring and antioxidant properties. All of them have been described as compounds that reduce Aβ accumulation, protect mitochondria from Aβ toxicity, restore mitochondrial function, and attenuate cognitive impairment in animal models of AD, possessing mitochondrial restoring and antioxidant properties [133].

Currently, nicotinamide (vitamin B3, NAM) and its derivatives are also under investigation. Vitamin 3 normalize redox levels and is also highly relevant for proper activity of Complex I, particularly NAD+ and NADH [134].

N-Methyl, N-propynyl-2-phenylethylamine (MPPE) is another drug candidate that has been assessed in animal models. MPPE prevents MPTP-induced nigral cell loss, upregulates mitochondrial superoxide dismutase to alleviate oxidative stress, and improves the function of Complex I [135]. 

### 5.2. Regulation of Mitochondrial Homeostasis and Inflammation

The promising strategy to ameliorate mitochondrial homeostasis in neurodegenerative disease is the induction of PGC-1a via the activation of PPAR receptors by rosiglitazone or bezafibrate, modulating PGC-1a activity by targeting 5′AMP-activated protein kinase (AMPK) employing 5-aminoimidazole-4-carboxamide ribotide (AICAR), metformin, or resveratrol. Recently, Wang et al. showed that resveratrol protected neurons against Aβ-induced disruption of spatial learning, memory, and synaptic plasticity [136]. In lymphoblastoid cell lines (LCLs) from AD patients, resveratrol increased the expression of gene encoding anti-aging factor sirtuin-1 (SIRT 1), the expression of which is decreased by the accumulation of Aβ [137].

Besides, the studies showing the neuroprotective effect of resveratrol against AD, several investigations were focused on its effects in experimental models of PD. In vitro studies indicated that resveratrol possesses neuroprotective properties against the damage produced by 6-OHDA, MPTT, and rotenone [138,139,140]. Moreover, this observation was supported by an animal study, which showed the neuroprotective effect of resveratrol against 6-OHDA, MPTP, and rotenone actions counteracting the motor and cognitive changes induced by these neurotoxins [141,142,143].

Recently, it has also been evaluated whether pharmacological agents and lifestyle interventions could improve mitochondrial homeostasis and enhance mitophagy. One of them is rapamycin, which is a selected inhibitor of mTORC1. This substance has been extensively shown to reduce cognitive decline in several mouse models of AD and AD-like dementia [144,145]. Rapamycin increased the expression of key regulators of autophagy (LC3, Atg5, Atg7, and Atg12). Other compounds with structures similar to resveratrol, RSVA314 and RSVA405, were found to inhibit mTOR activity and to stimulate the degradation of Aβ by the autophagic-lysosomal machinery [146].

As we described above, damaged mitochondria play a crucial role in regulating the pro-inflammatory response of the cell through activation of the inflammasome complex. Su and colleagues indicated that the fatty acid amide hydrolase inhibitor URB597 (URB) reduced the impairment of mitophagy and inhibited the NLRP3 inflammasome pathway [147]. Lonnemann and co-workers showed that OLT1177, a specific inhibitor of the NLRP3 inflammasome, ameliorated the phenotype of APP/PS1 mice, as evidenced by rescued spatial learning and memory [148].

The above data suggest that multiple agents/molecules targeting the mitochondrial-mitophagy-inflammasome axis could be promising candidates for future development therapeutics against neurodegenerative diseases.

## 6. Conclusions

**Figure 5:**
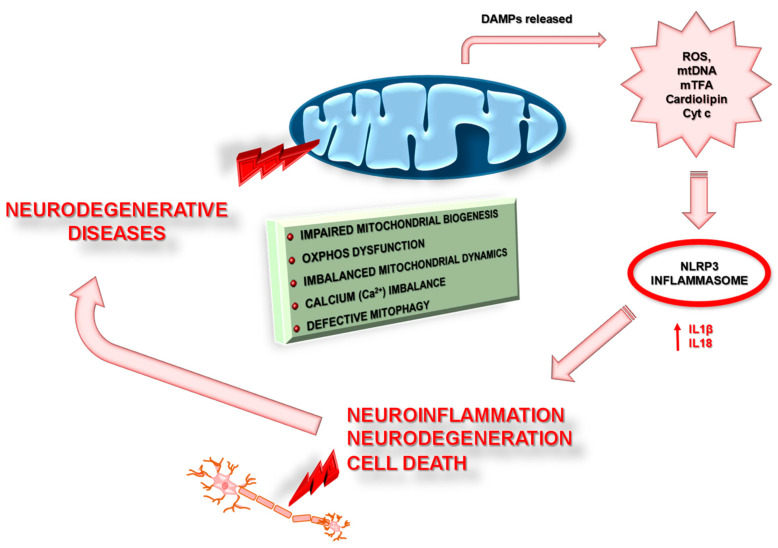
The potential mechanism linking mitochondrial dysfunction and activation of the NLRP3 inflammasome complex in neurodegenerative diseases. Mitochondrial dysfunction manifested by impaired mitochondrial biogenesis, OXPHOS (Oxidative Phosphorylation System) dysfunction, imbalanced mitochondrial dynamics and calcium, and defective mitophagy, which results in irreversible damage of mitochondria and the consequent release of its components—ROS (reactive oxygen species), mtDNA (mitochondrial DNA), mTFA (mitochondrial transcription factor A), membrane lipid cardiolipin, and proapoptotic proteins (including cytochrome c)—into the cytosol. Next, the leakage of mitochondrial components can act as intrinsic danger-associated molecular patterns (DAMP) and could activate the oligomerization domain-like receptor family members 3 (NLRP3 inflammasome complex), in turn boosting the production of cytokines such as Interleukin 18 (IL-18) and Interleukin 1β (IL-1β) and inducing neuroinflammation, neurodegeneration, and cell death.

In vitro, in vivo, and clinical studies have indicated that mitochondrial dysfunction plays a critical role in neuronal degeneration and disease progression in AD and PD. Data indisputably indicate that mitochondrial dysfunction in conjunction with NLRP3 activation contributes to the pathogenesis of neurodegenerative diseases, such as AD and PD. The mechanism is complex and interacts with other well-known pathomechanisms. Nevertheless, mitochondrial damage and the reduced ability of neurons to properly perform the mitophagy process causes the release of DAMPs from mitochondria, such as mtDNA, mTFA, cytochrome c, etc., which in turn may induce neuroinflammation and the progression of neurodegenerative diseases (Figure 5). A better understanding of the exact mechanisms by which DAMPs activate neuroinflammation will allow the introduction of new therapeutic strategies in the future.

## Data Availability

Not applicable.

## Abbreviations

AD	Alzheimer’s Disease
AIM2	Absent in Melanoma 2
ALS	Amyotrophic Lateral Sclerosis
AMPK	5′AMP-activated protein kinase
APOE4	ApolipoproteinaE-allele4
APP	Amyloid Precursor Protein
ASC	Adaptor Apoptosis Speck Protein
ATP	Adenosine Triphosphate
Aβ	β-amyloid
AβPP	Amyloid-β Protein Precursor
CL	Cardiolipin
CNS	Central Nervous System
COX	Cytochrome c oxidase
DA	Dopamine
DAMP	Damage-Associated Molecular Patterns
Drp1	Dynamin-Related GTPase
ERR-β	Estrogen-related receptors -β
ERR-γ	Estrogen-related receptors -γ
ERR-α	Estrogen-related receptors -α
ETC	Electron Transport Chains
Fis1	Mitochondrial fission 1 protein
HD	Huntington’s Disease
IL-18	Interleukin 18
IL-1β	Interleukin 1β
IL-6	Interleukin 6
IL-8	Interleukin 8
IMM	Inter Mitochondria Membrane
INF-γ	Interferon γ
LBs	Lewy bodies
LC3	Microtubule Associated Proteins 1A/1B light chain 3B
LOAD	Late-onset AD
MAP-5	Microtubule Associated Protein-5
Mff	Mitochondrial Fission Factor
MFN1	Mitofusin 1
MFN2	Mitofusin 2
Mid49	Mitochondrial Dynamics Proteins of 49
Mid51	Mitochondrial Dynamics Proteins of 51
Miro1	Mitochondrial Rho GTPase1
MPP+	1-methyl-4-phenylpyridinium
mPTP	Mitochondrial Permeability Transition Pore
MS	Multiple Sclerosis
mtDNA	Mitochondria DNA
mTFA	Mitochondrial Transcription Factor A
mtΔΨ	Mitochondrial Membrane Potential
nDNA	Nuclear DNA
NDs	Neurodegenerative Diseases
NFTs	Neurofibrillary Tangles
NF-κβ	Nuclear Factor kappa-light-chain-enhancer of activated β cells
NLRC4	CARD domain-containing protein 4/IPAF ICE-Protease Activating Factor
NLRP2	NOD-like receptor protein 2
NLRP3	NOD-like receptor protein 3
NRF1	Nuclear Respiratory Factor 1
NRF2	Nuclear Respiratory Factor 2
OMM	Outer Mitochondrial Membrane
OPA1	Optic Dominant Atrophy 1
OXPHOS	Mitochondrial Oxidative Phosphorylation System
PAMP	Pathogen-Associated Molecular Patterns
PD	Parkinson’s disease
PGC-1α	Peroxisome proliferator activated receptor gamma coactivator-1α
PINK1	PTEN-induced putative kinase 1
PRRs	Pattern Recognition Receptors
PS1	Presenilin-1
RNS	Reactive Nitrogen Species
ROS	Reactive Oxygen Species
rRNAs	Ribosomal RNAs
SN	Substantia Nigra
SNCA	α-synuclein
sPD	Sporadic Form of Parkinson’s Disease
SPs	Senile Plaques
TCA	Tricarboxylic Acid Cycle
TIM	Translocase of the Inner Membrane
TIM23	Translocase of the Inner Mitochondrial Membrane 23
TLRs	Toll-Like Receptors
TNF-α	Tumor Necrosis Factor α
TOM	Translocase of the Outer Membrane
TOM20	Translocase of Outer Membrane 20
Trem2	Triggering receptor expressed on myeloid Cells 2
TREM2	Triggering Receptor Expressed on Myeloid Cells 2
tRNAs	Transfer RNAs
VDAC1	Voltage-Dependent Anion-Selective Channel 1
αKGDH	Pyruvate Dehydrogenase Complex, α-Ketoglutarate Dehydrogenase
